# Neuromodulation with chemicals: Opportunities and challenges

**DOI:** 10.1016/j.fmre.2024.04.010

**Published:** 2024-04-12

**Authors:** Yifei Pan, Cong Pan, Lanqun Mao, Ping Yu

**Affiliations:** a Key Laboratory of Analytical Chemistry for Living Biosystems Institute of Chemistry, Chinese Academy of Sciences, Beijing 100190, China; b College of Chemistry, Beijing Normal University, Beijing 100875, China

**Keywords:** Neuromodulation, Chemical delivery, Brain chemistry, Neurochemistry, *In vivo*

## Abstract

Chemicals play a crucial role in neurophysiological and neuropathological processes. By regulating the concentration of specific chemicals, receptors on the neuron cell membrane can be modulated to activate or inhibit, thereby influencing specific ion channels and facilitating neuromodulation. This review introduces several chemical modulation techniques, such as microinjection, electrode/nanoparticle-based chemical delivery methods, *in situ* electrochemical synthesis and chemogenetics. While these techniques show promise in expanding the application of chemical neuromodulation, they currently exhibit different degrees of shortcomings and room for improvement. This review summarizes the opportunities and challenges for chemical neuromodulation methods and provide an outlook for their prospects in the future.

## 1 Introduction

The brain is the most sophisticated organ in the living system, and its complex and dynamically changing chemical composition contributes to its functions as the central nervous system (CNS). Thus, precise tools for recording and manipulating neurochemical signals are crucial for understanding the dynamic chemical changes in the brain during physiological and pathological processes. Currently, most neurological diseases, especially neurodegeneration diseases such as Parkinson’s disease (PD) and Alzheimer’s disease (AD), still necessitate further research into their mechanisms and therapies. Therefore, there is an urgent need to develop technologies capable of regulating neural activities with high temporal-spatial resolution. Neuromodulation is crucial for elucidating cell function in fundamental research and for treating brain-related disorders [1].

**Fig. 1:**
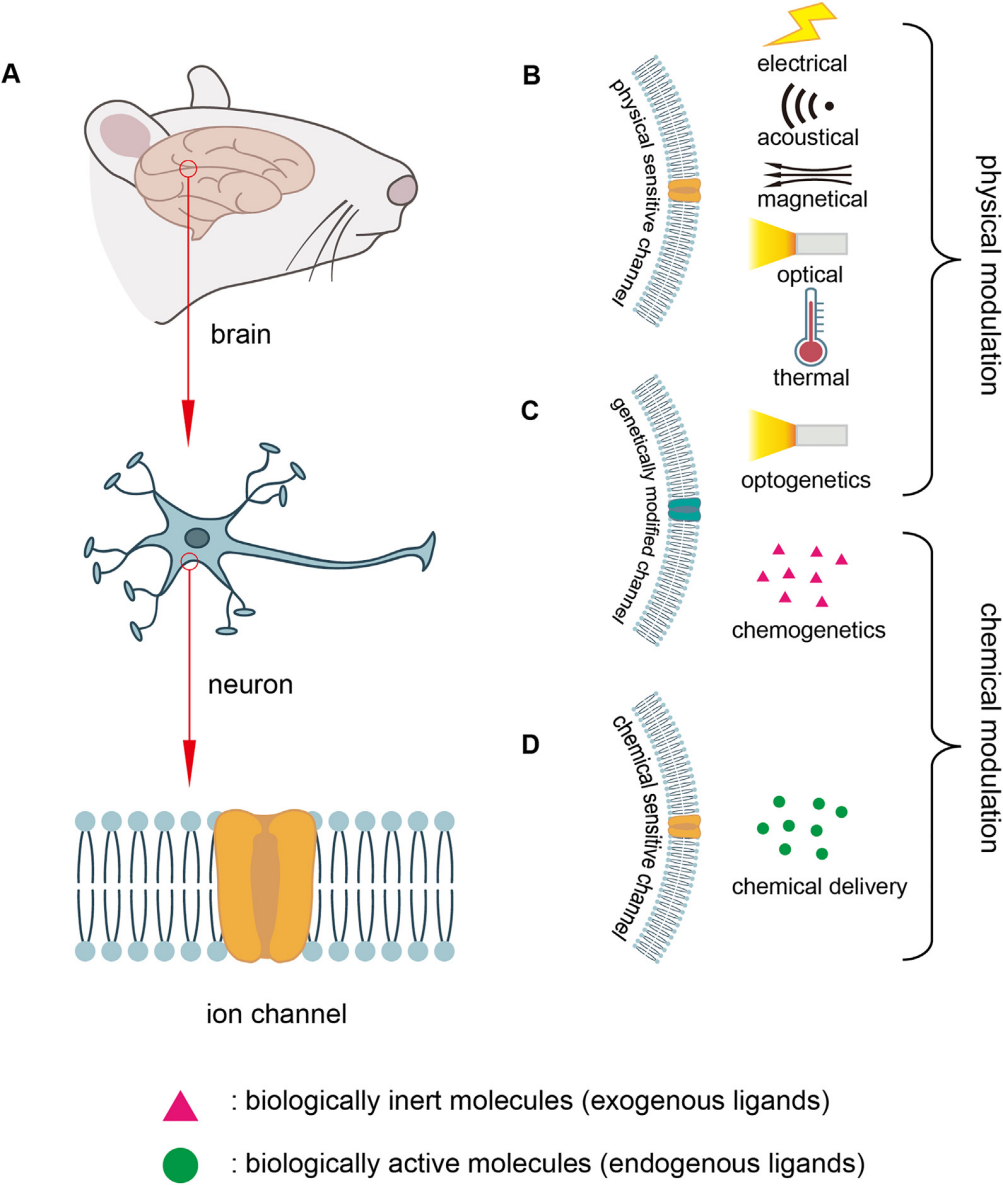
**Mechanisms and technologies of neuromodulation.** (A) Three scales for studying neural activities: brain, neuron, ion channel on the membrane. (B) Physical modulation including electrical, magnetical, acoustical, optical, thermal methods. (C) Modulation involving genetical modification: optogenetics for physical, chemogenetics for chemical. (D) Chemical delivery using neural active molecules.

The brain consists of heterogeneous and sophisticated ensembles of neurons (Fig. 1A). Nerve electrical signals originate from the rapidly changing membrane potential of neurons [2], [3], [4]. Signal transmission in the brain corresponding to various senses relies on the ability of neurons to respond to slight stimuli by rapidly changing the membrane potential across cells [5], [6], [7]. Individual sensory cells produce changes in membrane potential in response to slight stimuli. For example, receptors in the eye respond to the light of a single photon [8], [9], [10], olfactory neurons detect a single odor molecule [11,12], and hair cells in the inner ear respond to minuscule atomic-scale movements [13]. These rapid changes in membrane potential are mediated by ion channels, a class of integral membrane proteins present in all cells. The ion channels of neuron cells are finely tuned for rapid information processing. Meanwhile, they are heterogeneous among neurons, allowing for specific signal transmission in different parts of the nervous system via distinct types of channels [14], [15], [16].

Signal transmission during brain functions is simultaneously modulated by complex electrical and chemical signals [17]. Various neurochemicals mediate neural physiological and pathological processes. Chemical signaling between synapses is the most common and important pathway in CNS. Specifically, once neuronal electrical signals are transmitted to the axon terminal, neurotransmitters inside the synapse of the pre-synaptic membrane are secreted and released into the synaptic cleft through vesicles. These neurotransmitters then bind to receptor proteins on the post-synaptic membrane, activating subsequent neurons to generate action potentials and completing inter-neuronal signaling [18]. In the brain, various neurotransmitters (e.g., acetylcholine, dopamine, glutamate, and gamma-aminobutyric acid) are produced, transported, and converted by synapses in neural systems, performing essential functions [19]. For instance, dopamine plays a central role in reward mechanisms [20] while 5-hydroxytryptamine is involved in thermoregulation [21]. Additionally, neuromodulators, such as ascorbic acid [22], ions [23], amino acids [19], and peptides [24], perform corresponding biological functions in the brain. The regular operation of brain function relies on the precise spatiotemporal modulation of specific neurons by various neurochemicals in the brain. Contrarily, irregular changes in neurochemicals result in serious neurological disorders or even brain diseases such as PD, AD, and depression [1,25]. Therefore, neuromodulation of chemical molecules in the CNS is of great necessity, and several obstacles exist to be overcome [26].

Currently, physical modulation methods such as electrical stimulation, magnetic stimulation, focused ultrasound stimulation, optical modulation, and thermal modulation methods have been utilized for neuronal activation and projection of neural circuits (Fig. 1B). Apart from these methods, genetical modifications of channels have been investigated for higher selectivity methods of modulation (Fig. 1C). To date, optogenetics and chemogenetics have emerged as the most commonly utilized physical and chemical technologies, respectively. Unlike chemogenetics, which utilizes designed exogenous ligands to bind to specific modified receptors, chemical delivery methods using biologically active compounds to precisely stimulate neurons in specific brain regions and modulate their activity remain challenging (Fig. 1D) [27]. Generally, physical modulation methods offer higher temporal resolution, but their precision and specificity are low and uncontrollable. Moreover, physical methods may entail certain side effects, such as electrical interference, heat injury, and illumination injury. Compared to physical modulation, chemical modulation employs receptor-targeted chemicals that can activate or inhibit the specific ion channels in specific brain regions, providing greater specificity and traceability. Furthermore, unlike common delivery methods (oral, nasal, subcutaneous, intraperitoneal, and intravenous), intracerebral chemical delivery methods bypass metabolic processes and the blood-brain barrier, offering a more rapid and adequate response. In this review, we introduce several strategies for chemical-induced neuromodulation including microinjection, electrode/nanoparticle-based chemical delivery, *in situ* electrochemical synthesis, and chemogenetics.

## 2 Microinjection

The stable chemical environment in the brain is significant for the regular activities of neurons. Specific chemical changes can either excite or inhibit corresponding neural activities. Thus, delivering neuroactive chemicals into the nervous system via controllable methods is proposed as an effective approach for neuromodulation.

Microinjection is a simple and efficient strategy to deliver chemicals in the brain. Usually, animals are surgically implanted with a guide cannula over the specific brain region. Following recovery from surgery, an internal cannula is inserted through the guide cannula into the specific brain region of the experimental animal. Using a specialized syringe pump capable of accurately delivering very small volumes (typically less than 0.5 µL), drug-containing solutions are injected directly into the brain of the experimental animal. A slow delivery rate (typically 0.1 to 0.5 µL/min) ensures that pressure from the microinjection does not damage or displace the implanted brain tissue. Subsequently, the injected solutions diffuse away from the tip of the internal cannula to the specific brain region, as much as 1 mm away for a 0.5-µL volume [28].

Microinjection enables researchers to study and manipulate systematically at molecular level within a specific brain region. These delivered molecules typically include neuroactive chemicals, such as neurotransmitters, neuromodulators and ions. For example, microinjection of noradrenaline (NE) into the dorsal periaqueductal gray area (dPAG) causes a pressor response mediated by vasopressin release into the circulation. The synapses within the diagonal band of Broca (dbB) are involved in the pressor pathway and activated by the microinjection of NE. By microinjection, direct neural projection from the dPAG to the dbB was proposed to constitute the neuroanatomic substrate for pressor pathway [29]. Moreover, microinjection of NE into the medial amygdaloid nucleus (MeA) also induces pressor and bradycardiac response mediated by magnocellular neurons in both the paraventricular and supraoptic of the hypothalamus [30]. Chemical stimulation over the periaqueductal grey matter (PAG) by microinjecting D,L-homocysteic acid (DLH) to preferentially activate somatodendritic receptors decreased the frequency of micturition. Because DLH is an amino acid that mimics the action of an excitatory neurotransmitter, so neurons in the critical relay area are depolarized and neurotransmissions are facilitated by microinjection of DLH [31]. Microinjection of orphanin FQ (OFQ) into periaqueductal gray affect sensory processing in the wide dynamic range neurons of the spinal dorsal horn for pain modulation [32]. Apart from neurotransmitters that mediate synaptic signaling directly, injection of certain receptor agonists or antagonists also regulates neuron signals due to their effects on corresponding receptors. For example, under sevoflurane anesthesia, an injection of orexin receptor agonist (orexin-A or orexin-B) in basal forebrain (BF) decreases the emergence time, whereas an injection of orexin receptor antagonist (SB-334867A) delays the emergence time [33]. Microinjection of the cholinergic agonist carbachol into the bed nucleus of the stria terminalis (BST) has been reported to cause pressor response in unanesthetized rats, which is mediated by an acute release of vasopressin into the systemic circulation, followed by baroreflex-mediated bradycardia [34]. Microinjection of GABA_A_-receptor agonists into an upper brainstem region, mesopontine tegmental anesthesia area (MPTA), induces rats to an anesthesia-like state [35]. Microinjection of propofol into the perifornical area decreases cortical acetylcholine release, thus inducing sedation in rats [36]. Microinjection of *p*-chlorophenylalanine (PCPA) into putative serotonergic dorsal raphe nucleus (DRN) inhibits the synthesis of serotonin (5-HT) and causes insomnia [37].

**Fig. 2:**
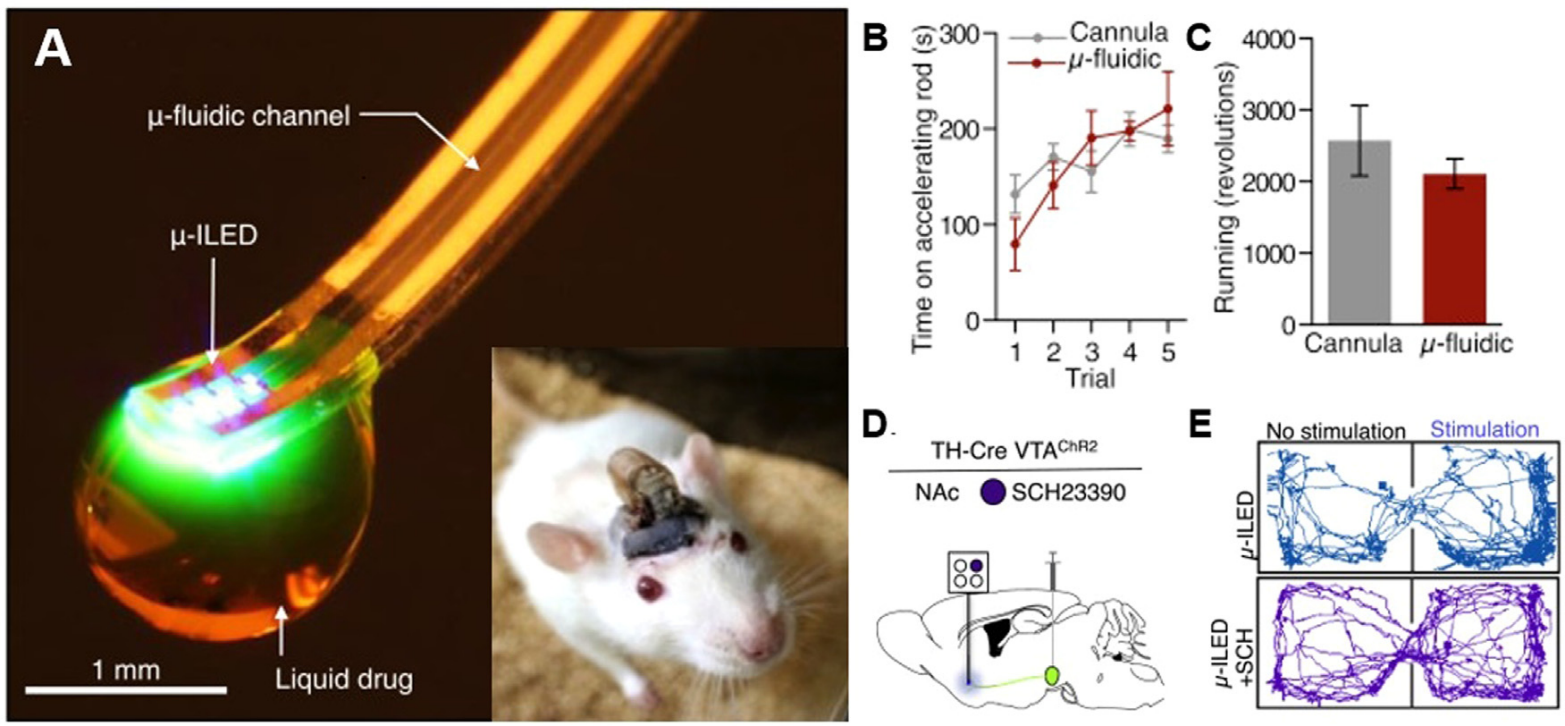
**Wireless microfluidic neural probe for *in vivo* pharmacology and optogenetics.** (A) An optical image of the flexible optofluidic probe consisting of multiple microfluidic channels and µ-ILEDs, capable of simultaneous pharmacological delivery and optical stimulation. (B, C) Activity profiles of mice implanted with wireless- and cannula-based devices. (D) A schematic diagram illustrating the wireless optofluidic experiment. (E) Traces of mice during real-time place preference control using wireless optofluidics [39].

Recent studies have reported on the development of wireless injectable microfluidics for chemical neuromodulation. Although metal cannulas have been utilized for pharmacological infusions, they lack the spatial precision for brain-region scale and are unsuitable for long-term clinical intervention. This technology lacks the ability to control drug delivery at the cellular level, and it also lacks the multifunctionality required to support wireless, simultaneous optical and electrical neural stimulation and recording. To overcome these limitations, innovative efforts have combined neuroscience with engineering to develop spatiotemporally-precise tools for *in vivo* neuropharmacology, such as microfluidic neural probe systems. Microfluidic neural probe systems significantly reduce damage to neural tissue, enable the delivery of multiple distinct pharmacological or fluid agents, and facilitate integration with various other modalities, such as optical, electrical, and chemical components, within a single implant [38], [39], [40]. Fig. 2 shows a wireless microfluidic system that incorporates optoelectronics for *in vivo* pharmacology and optogenetics [39]. One of the most advanced features of the wireless optofluidic system is its ability to simultaneously implement wireless *in vivo* optical stimulation (e.g. for optogenetics) and pharmacology. In a demonstration experiment, optical stimulation of ventral tegmental area (VTA) dopaminergic neurons induced a real-time place preference, while concurrent wireless delivery of a dopamine receptor-1 antagonist (SCH23390) blocked this behavior. Such wireless design is desirable to enable unprecedented cell-type and receptor-selective circuit manipulation in freely moving animals.

Overall, microinjection serves as a simple and efficient tool for neuromodulation, allowing direct injection of chemicals into specific brain regions. However, the diffusion of microinjected chemicals occurs over the span of minutes, thereby limiting the temporal resolution. Moreover, the diffusion of microinjected chemicals away from the delivery point complicates the delivery of a desired concentration in the targeted brain region.

## 3 Electrode-based chemical delivery

**Fig. 3:**
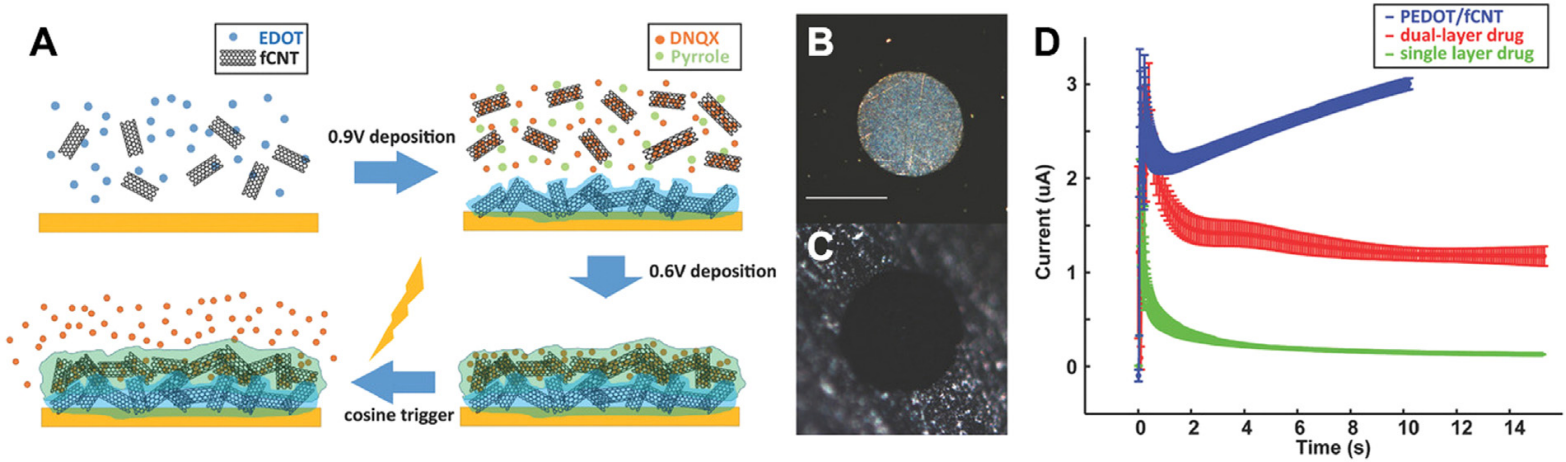
(A) Illustration of the synthesis of dual-layer PEDOT/fCNT-PPy/fCNT/DNQX film and controlled release of DNQX from the film. (B) microscope examination of custom-built in vitro microelectrode, scale bar = 100 µm. (C) picture of PEDOT/fCNT-coated in vitro microelectrode site. (D) chronoamperometry deposition of PEDOT/fCNT (*n* = 4), dual-layer PPy/fCNT/fluorescein (*n* = 4), and single-layer PPy/fCNT/fluorescein (*n* = 3) [48].

Microelectrodes are frequently used for *in vivo* neural recording and electrochemical modulation of neural microcircuit activity [41]. Besides, coated microelectrodes that load chemicals offer a strategy for delivering chemicals to targeted brain regions. Conducting polymers have been selectively grown on individual microelectrodes to increase electrical conductivity by ionic dopants. This allows the conducting polymer-electrode to repeatedly release small quantities of doping molecules into the solution upon electrical stimulation [42], [43], [44], [45], [46]. When applied to multielectrode arrays (MEAs), this technique enables the controlled release of pre-incorporated neurochemicals from the given electrode or multiple electrodes simultaneously during neuronal recording. Cui et al. [47] developed rapid neuronal activity modulation strategy involving the controlled release of neurochemicals from polypyrrole (PPy) coated microelectrodes. By applying brief voltage pulses that electrochemically reduce the polymer, the pre-incorporated 6-cyano-7-nitroquinoxaline-2,3-dione (CNQX), an inhibitor of AMPA-type glutamate receptors, dissociates and diffuses away, achieving locally effective concentrations at last. Subsequently, inhibition of evoked synaptic currents in neurons was observed, and spiking activity of neurons in local circuits recorded extracellularly near the releasing electrode was silenced for a similar duration following release. This work was further optimized by utilizing 6,7-dinitroquinoxaline-2,3-dione (DNQX) instead of CNQX due to its higher water solubility. Additionally, by adding functionalized carbon nanotubes(fCNT) into PPy and poly(3,4-ethylenedioxythiophene) (PEDOT) films, the electrical conductivity and mechanical stability of the conducting polymer films were improved. The dual-layer coating is capable of both loading and electrically releasing DNQX, thus inducing effective neural inhibition [48] (Fig. 3).

## 4 Nanoparticle-based chemical delivery

Motivated by recent advancements in materials science and nanotechnology, various nanomaterials have been developed for neuromodulation. Functional particles with small sizes, tailored architectures and therapeutic motifs that facilitate targeted drug delivery are widely engineered. These particles are constructed using a wide range of substrates, including organic (e.g., liposomes, micelles, hydrogels) [49], [50], [51], inorganic (e.g., metal/metal oxide particles, silica nanoparticles, quantum dots, etc.) [52,53], and biomass-derived materials (e.g., exosomes, cells, bacteria, etc.) [54], [55], [56]. Therefore, nanoparticles can serve as an ideal carrier of neuroactive chemicals to modulate neural circuits.

**Fig. 4:**
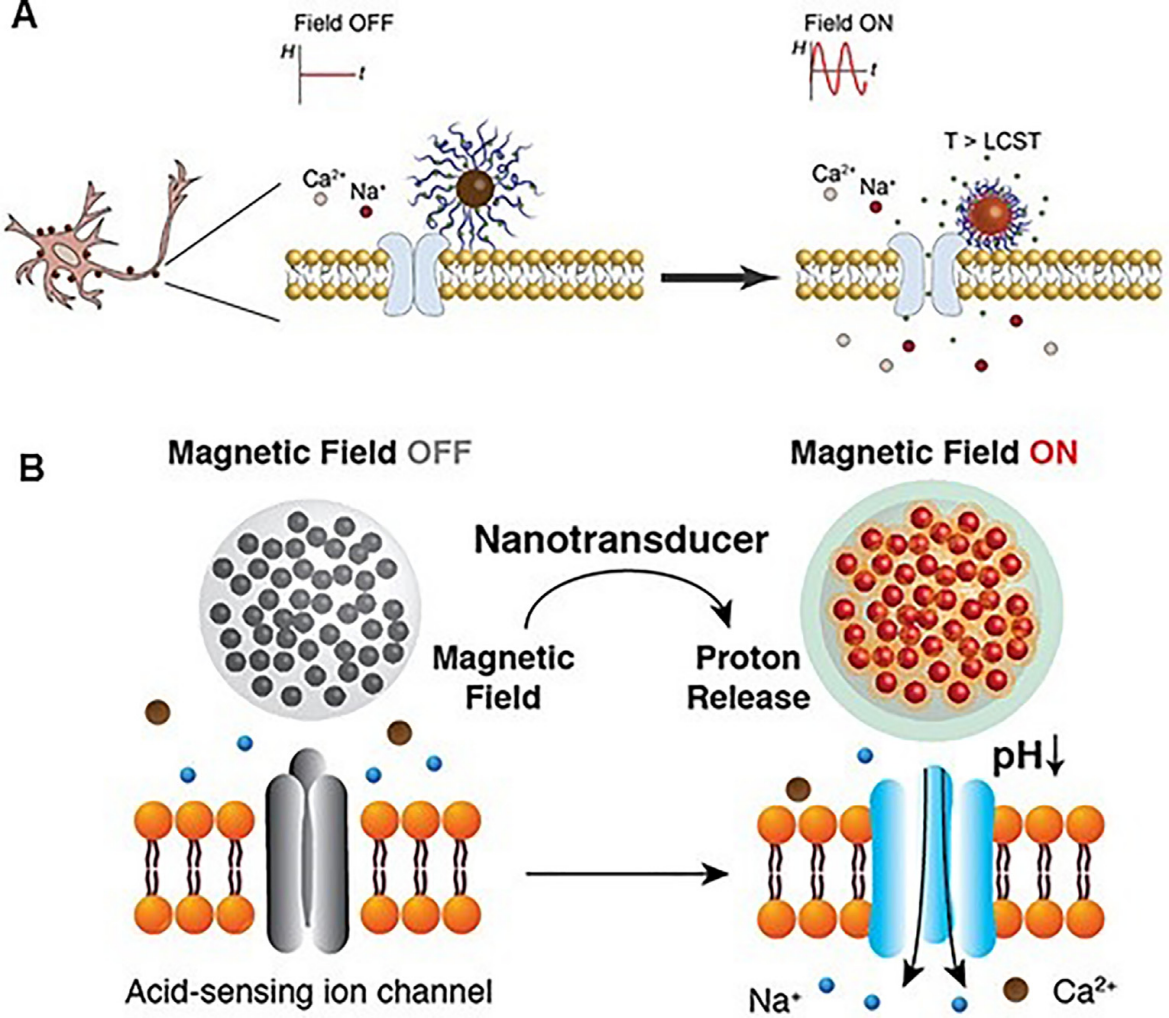
(A) An overview schematic diagram of the chemomagnetic neuromodulation approach. Upon exposure to AMF, the heat dissipated by magnetic hysteresis increases the local temperature reaching the low critical solution temperature (LCST) of the polymer. At the LCST the thermodynamic phase transition of POEGMA brushes coating on MNPs surface initiates, causing polymer collapse and releasing dopamine as a result. Dopamine release stimulates D1 and D2 receptors on striatal neurons allowing for intracellular ion influx. [57] (B) Illustration of the wireless proton generating system. MNP heating elevates temperature inside the nanotransducers and accelerates the hydrolysis of polyanhydride and polyester. The carboxyl groups generated during the hydrolysis reduce the extracellular pH and activate neurons by triggering ASICs [58].

Signals of neuron cells can be chemically regulated by nanoparticles. Romero et al. [57] utilized iron oxide magnetic nanoparticles (MNPs) that were functionally coated with thermoresponsive poly (oligo (ethylene glycol) methyl ether methacrylate) (POEGMA) brushes loaded with dopamine (Fig. 4A). Dopamine-loaded MNPs-POEGMA are co-cultured with primary striatal neurons. When exposed to alternating magnetic fields (AMFs), MNPs undergo hysteresis power loss and dissipate heat. The local heat produced by MNPs initiates a thermodynamic phase transition on POEGMA brushes resulting in polymer collapse and dopamine release. AMF-triggered dopamine release enhances the response of dopamine ion channels expressed on the cell membranes enhancing the activity of approximately 50% of striatal neurons subjected to the treatment. Anikeeva et al. [58] designed nanotransducers capable of converting AMF into protons in physiological environments (Fig. 4B). When magnetic MNPs are exposed to AMFs, the heat dissipated triggers a hydrolytic degradation of surrounding polyanhydride or polyester, releasing protons into the extracellular space. Remote magnetic control of local protons has been shown to trigger acid-sensing ion channels and evoke intracellular calcium influx in hippocampal neurons.

Directly injected nanoparticles can also be utilized to release chemicals for neuromodulation in the brain. Anikeeva et al. [59] reported a neuromodulation technique based on MNPs that release chemical payloads in the presence of AFMs. The heat dissipated by magnetic nanoparticles in the presence of AMFs triggers the release of chemical neuromodulators from thermally sensitive lipid vesicles, with a 20-second latency. Injection of the magnetoliposomes into the ventral tegmental area (VTA) followed by AMF stimulus induces the remote modulation of motivated behavior in mice.

Although neuromodulation through nanoparticles drug delivery system has proven to be effective, addressing potential infections or inflammation within the brain remains a challenge. Therefore, non-invasive neuromodulation techniques based on nanoparticles for chemical delivery across blood–brain barrier (BBB) are essential. Green et al. [60] developed nanoparticles that allow noninvasive uncaging of a neuromodulatory drug, in this case the small molecule anesthetic propofol, upon the application of focused ultrasound (FUS). After an intravenous infusion of the nanoparticles, FUS were applied on the rat brain. Subsequently, these particles released propofol and silenced seizures in an acute rat seizure model without brain parenchymal damage or blood-brain barrier opening. Zhao et al. [61] reported a delivery pathway based on Vitamin B1 (VB1) incorporated MIL-101-NH_2_(Fe) nanoparticles containing pralidoxime chloride (2-PAM). The composite drug could effectively cross the blood-brain barrier and restore the acetylcholinesterase (AChE) activity in the brain of poisoned mice.

## 5 In situ electrochemical synthesis

**Fig. 5:**
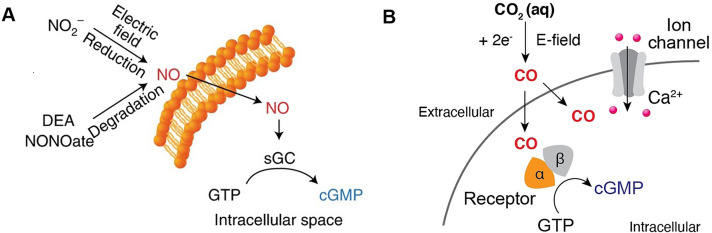
(A) Illustration of the NO-sGC-cGMP signaling pathway in genetically intact cerebellar neurons [62]. (B) Schematic illustrating activation of sGC mediated by electrochemically produced CO [64]. (GTP: guanosine 5′ triphosphate)

All the techniques reviewed above focus on delivering exogenous compounds. However, repeated delivery is required once the drugs are ingested. Recently, *in situ* electrochemical synthesis of neuroactive compounds has been investigated to solve the problem. Anikeeva et al. [62] developed an iron sulfide nanocluster that catalyze nitric oxide generation from benign sodium nitrite in the presence of modest electric fields for *in situ* electrochemical synthesis of NO and real-time neuromodulation. (Fig. 5A) Locally generated nitric oxide activates the nitric oxide-sensitive cation channel, transient receptor potential vanilloid family member 1 (TRPV1), and TRPV1-mediated Ca^2+^ responses can be controlled by varying the applied voltage. Integrating these electrocatalytic nanoclusters with multimaterial fibers allows NO-mediated neuronal interrogation *in vivo*. The *in situ* generation of NO in the ventral tegmental area with the electrocatalytic fibers evoked neuronal excitation in the targeted brain region and its excitatory projections.

Other attempts have been carried out on neural signaling modulation in single-cell model. Eric et al. [63] achieved localized peroxide delivery using conducting polymer (poly(3,4-ethylenedioxythiophene)(PEDOT)), and selectively catalyzed the oxygen reduction reaction to produce hydrogen peroxide (H_2_O_2_). The electrochemically-delivered H_2_O_2_ was reported to modulate human H_2_O_2_-sensitive Kv7.2/7.3 (M-type) channels expressed in a single-cell model using Xenopus laevis oocytes. Anikeeva et al. [64] developed an electrochemical approach that affords on-demand release of CO through reduction of carbon dioxide (CO_2_) dissolved in the extracellular space. (Fig. 5B) Electrocatalytic generation of CO by cobalt phthalocyanine molecular catalysts modulates signaling pathways mediated by a CO receptor soluble guanylyl cyclase. The electrochemical microscale fibers were able to produce CO in a spatially-restricted manner and activate signaling cascades in hippocampal neurons.

## 6 Chemogenetics

**Fig. 6:**
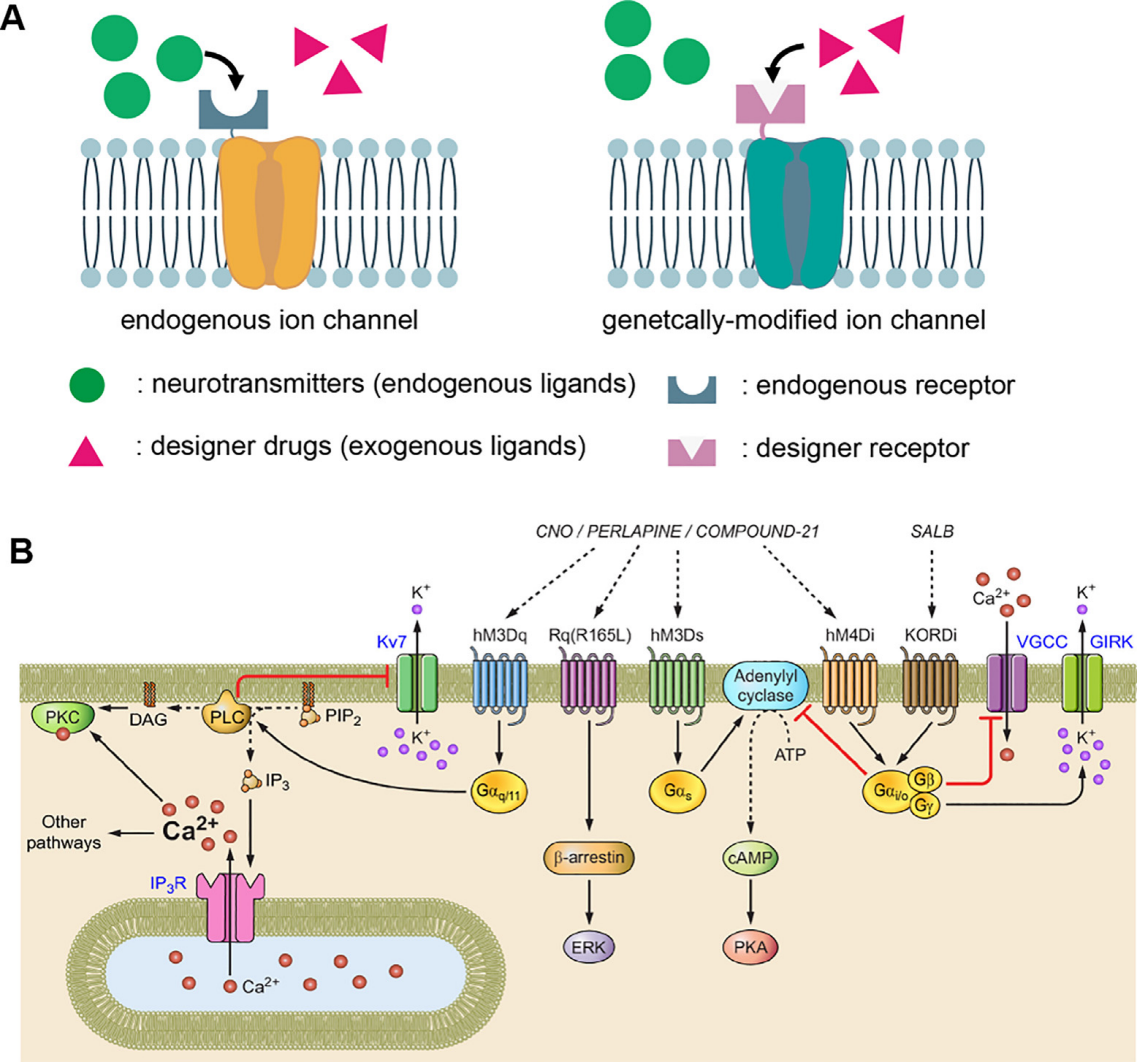
(A) Illustration of the working principle of chemogenetics. (B) DREADDs and their downstream intracellular signal transduction pathways [75].

In recent years, chemogenetics has emerged as an alternative technique to optogenetics, replacing optics with pharmacology to modulate neurological processes. Chemogenetics is defined as the modulation of neural activity through neurotransmitter receptors that are genetically engineered to bind specific exogenous ligands. In this regard, it represents an inversion of the normal method of pharmacology that designs ligands to bind specific receptors. (Fig. 6) The use of chemogenetics was spearheaded after the introduction of Designer Receptors Exclusively Activated by a Designer Drug (DREADDs) in 2007 by the Roth lab [65]. DREADD is an umbrella term encompassing a group of genetically engineered G protein-coupled receptors (GPCRs) with altered ligand responsiveness. DREADDs are unresponsive to their native, endogenous ligands, instead, they are exclusively activated by engineered drugs [66]. For example, the excitatory designer receptors (such as hM3Dq) binding of clozapine-N-oxide (CNO) ultimately produces neuronal burst firing by increasing intracellular calcium levels; the inhibitory designer receptors (such as hM4Di) binding of CNO silences neuronal activity by inhibiting adenylate cyclase [66]. To achieve activation and inhibition of the same neuronal populations with different ligands in the same animal, inhibitory κ-opioid receptor-based DREADDs have been developed [67]. By binding the designer ligand salvinorin B to κ-opioid receptor, DREADDs also inhibits adenylate cyclase, leading to neuronal inhibition. When κ-opioid receptor–based DREADDs are combined with excitatory hM3Dq receptors, clozapine-N-oxide can be used to excite the same neurons that are inhibited by salvinorin B. Chemogenetic technology has been used to engineer several different protein classes, including G protein-coupled receptors [65,[67], [68], [69], ligand-gated ion channels [70,71], kinases [72,73], and nonkinase enzymes [74].

Although chemogenetics offers powerful ways to probe neural circuit function by selectively manipulating neuronal activity patterns, there are still limitations to its reliability. These limitations include off-target effects (e.g., activation/inhibition of non-targeted cells), incomplete penetrance (i.e., not all targeted cells respond equally well), compensatory mechanisms (e.g., homeostatic plasticity), and others. Therefore, it is important to carefully consider these factors when interpreting data obtained from chemogenetics. Techniques involving genetic modification, including chemogenetics and optogenetics, can only be used on non-primate animals, and are not suitable for therapy of nerve diseases. In summary, chemogenetics has been widely used as a research tool in neuroscience and applied to animal models, but its clinical translation is hindered by ethical concerns and technical limitations. Further efforts are needed to address the off-target effects and improve the safety of this technique.

## 7 Summary and future prospects

Chemicals play an important role in neurophysiological and neuropathological processes. By regulating the concentration of specific chemicals applied, receptors on the neuron cell membrane can be modulated to activate or inhibit, thereby affecting specific ion channels and acheving neuromodulation. In this review, several techniques involving chemical modulation have been introduced including microinjection, electrochemical/nanoparticle induced drug delivery system, *in situ* electrochemical synthesis and chemogenetics. The advantages, limitations, chemicals and applications of each strategy have been summarized in Table 1.

**Table 1:** **Systematic comparison of chemical neuromodulation strategies**.

Strategy	Advantages	Limitations	Chemicals & Applications
Microinjection	• Technologically sophisticated • Non-genetical modification	• Temporal resolution at minute level • Uncontrollable concentration	• NE, pressor response [29,30] • DLH, micturition frequency decrease [31] • OFQ, pain modulation [32] • Propofol, sedation [36]
Electrode-based chemical delivery	• Release-controllable • Integration of record and regulation • Non-genetical modification	• Invasive implantation • Nonrenewable	• CNQX/DNQX, neural inhibition [47,48]
Nanoparticle-based chemical delivery	• Remote modulation • Non-genetical modification	• Infection or inflammation • Nonrenewable	• Proton/dopamine, neuron activation [57,58]
*In situ* electrochemical synthesis	• Controllable interface • High selectivity • Non-genetical modification	• Side reactions	• NO/H_2_O_2_/CO, neuron activation [62], [63], [64]
Chemogenetics	• Less invasive • High specificity	• Genetical modification • Off-target effects • Incomplete penetrance • Compensatory mechanisms	• CNO, neuron activation/inhibition [66,67]

Chemogenetics involves genetic modification and is therefore unsuitable for the therapy of nerve diseases. Moreover, off-target effects, incomplete penetrance, and compensatory mechanisms are challenges that must be addressed to enhance the reliability of chemogenetics. Nevertheless, chemogenetics offers a valuable tool for investigating neural circuits and manipulating neuronal activity patterns.

Microinjection and electrode/nanoparticle-based chemical delivery methods are used to import exogenous compounds to specific brain regions. However, repeated administration is necessary once the drugs are ingested, which is nonrenewable. Local concentration depends on chemical diffusion, resulting in an uneven and uncontrollable distribution. Additionally, the temporal resolution is relatively low, measured at the minute level. Furthermore, the invasive implantation may cause infection or inflammation. Further efforts are required to enhance biocompatibility and delivery efficiency by investigating more biocompatible materials and diffusive neuroactive chemicals.

*In situ* electrochemical synthesis indirectly regulates chemical concentration by generating mediated chemicals that can be activated by electrochemical methods. The controllable interface and high selectivity show an excellent performance of neuromodulation. However, side reactions during the modulation process may lead to suboptimal results and reduced catalytic efficiency. The development of more sophisticated catalytic pathways and improved catalysts holds promise for expanding the applications for *in situ* electrochemical neuromodulation.

Chemical neuromodulation has undergone significant development. Chemistry plays a crucial role in enhancing drug delivery efficiency through the design of molecular-level interface structures, optimization of catalytic conditions, and mitigation of side reactions. The specificity and traceability of chemical modulation facilitate neuromodulation across neural pathways. Moreover, researchers continue to encounter challenges such as precise modification of neural interfaces, designing multiple molecules, fabricating novel materials, and implementing programmable control. The investigation of chemical neuromodulation is a multidisciplinary field that involves medicine, bioengineering, programming, and wireless technology. This field has established a platform that integrates physiological recording with an online intelligent modulation system. This platform provides the possibility for clinical experiments on neuromodulation and promotes the improvement of neuroscience therapy.

## Declaration of competing interest

The authors declare that they have no conflicts of interest in this work.
